# Contribution of animal models to the mechanistic understanding of Alternative Pathway and Amplification Loop (AP/AL)‐driven Complement‐mediated Diseases

**DOI:** 10.1111/imr.13141

**Published:** 2022-10-06

**Authors:** Beth G. Gibson, Thomas E. Cox, Kevin J. Marchbank

**Affiliations:** ^1^ Complement Therapeutics Research Group and Newcastle University Translational and Clinical Research Institute Faculty of Medical Science Newcastle‐upon‐Tyne UK; ^2^ National Renal Complement Therapeutics Centre aHUS Service Newcastle upon Tyne UK

**Keywords:** age‐related macular degeneration, alternative pathway, animal model, C3 glomerulopathy, complement, transgenic/knockout

## Abstract

This review aimed to capture the key findings that animal models have provided around the role of the alternative pathway and amplification loop (AP/AL) in disease. Animal models, particularly mouse models, have been incredibly useful to define the role of complement and the alternative pathway in health and disease; for instance, the use of cobra venom factor and depletion of C3 provided the initial insight that complement was essential to generate an appropriate adaptive immune response. The development of knockout mice have further underlined the importance of the AP/AL in disease, with the FH knockout mouse paving the way for the first anti‐complement drugs. The impact from the development of FB, properdin, and C3 knockout mice closely follows this in terms of mechanistic understanding in disease. Indeed, our current understanding that complement plays a role in most conditions at one level or another is rooted in many of these in vivo studies. That C3, in particular, has roles beyond the obvious in innate and adaptive immunity, normal physiology, and cellular functions, with or without other recognized AP components, we would argue, only extends the reach of this arm of the complement system. Humanized mouse models also continue to play their part. Here, we argue that the animal models developed over the last few decades have truly helped define the role of the AP/AL in disease.

## 1 INTRODUCTION

### 1.1 Cobra venom factor

A model is “just a model” and comparing across species is fraught with difficulty. However, we would argue the data generated in animal models is incredibly useful and, when used wisely, answers key questions. Thus, we will begin our section on the AP/AL of complement in animal models by directing you to the ground‐breaking work by Mark Pepys^1^, ^2^, ^3^, ^4^ and colleagues; a body of work that demonstrates the inexorable link between innate and adaptive immunity. These studies helped define the power of animal models to interrogate immunological mechanisms through perturbation of the controls on the AP/AL. We will not dwell too long on the findings of those studies here, but it felt important from a context perspective to discuss the use of cobra venom factor (CVF) in animal models (see Table 1). Of course, cobra venom has been used since the dawn of complement research to gain mechanistic insight into complement.^37^, ^38^ That said, it was undoubtedly in the 1960s and 70s that use of cobra venom came into its own.^39^, ^40^ It was quickly established that CVF activates and rapidly depletes complement activity. Since then, CVF has been a “go‐to” reagent for both in vitro and in vivo research and, without it, our understanding of the role of complement in many diseases would be significantly diminished.^41^ Professor Peter Lachmann and colleagues established that CVFBb is completely resistant to the regulatory actions of FH and FI.^42^ Of course, the natural beauty of CVF, honed over millennia, is that it works across a wide range of species. These properties make CVF a highly useful agent for the researcher. Indeed, the impressive ability of CVF to deplete C3 from animals led to the development of a humanized CVF for potential therapeutic applications.^43^ CVF consumes FB, C3 and partially reduces the levels of terminal pathway components, resulting in a loss of complement activity (including lytic activity) in an animal,^44^, ^45^ in a matter of minutes, and takes days for complement activity to return (as components are synthesized). This of course gives the researcher ample time to assess complement function in the short to medium term (please see Table 1) but it presents limitations for looking at loss of complement function both in a silent manner and over a protracted period. Indeed, repeated exposure to CVF reduces its efficacy due to the production of neutralizing antibodies; of which mice develop after a single exposure.^45^, ^46^ One of the interesting findings to come from the use of CVF in mice was that even in the absence of FD, CVFB was cleaved, albeit at a significantly slower rate,^47^ suggesting that CVF was able to transform FB into an active conformation in the absence of FD.^48^ A CVF transgenic mouse has also been generated, and while C3 levels were reduced to around 10‐20% of normal levels, the mice appeared healthy^49^ suggesting that low expression of C3 allows for most processes to continue unimpeded. For complete depletion of function, it is here that genetic knockout of complement proteins come into their own, and it is here that we shall focus this review.

**TABLE 1:** Use of CVF to illuminate the role of the AP/AL in disease

Disease/condition	Route	Model	Finding	Reference
Immune response	200U in 4 IP injections over 24 h	B/c; immune response to Antigen (SRBC, Ova, PVP360)	Complement important for T‐dependent response	1
IC handling	6U IV at 8 h intervals over 24 h	HRP/anti‐HRP	Complement important for immune complex formation and binding to Macrophage and FDC	5
Burn injury	200 U/kg as single dose 3.5 h preburn	Male B6 or CD‐1—placed in water bath, 80°C, for 30 s	Loss of the AP/AL protects mice from death after burn	6
Trypanosoma/Parasite clearance	0.5 mg IV 1 h before parasite introduced	Clearance of trypanosomes (*T.lewisi*)	Complement important for control of infection	7
Arthritic model	8U IP	B/c; peptidoglycan immunization	Complement for early stages of arthritis	8
IgA Nephropathy	N/A	B/c; oral and IV immunogen	Complement important for hematuria and glomerular damage in IgA Nephropathy	9
IC nephritis (glomerulopathy)	N/A	DBA/2J and B10.D2oSnJ	glomerular injury is dependent on complement components generated via C3	10
Extravascular clearance of RBC	N/A	B/c—antibody coated erythrocytes	Complement plays key role in early clearance of Ab coated E	11
Mycobacterial Lesion	10 μg IV 1 h or 8 h	B6; *m.vaccae—*the local Shwartzman reaction	Depletion of complement restricts inflammation in the model	12
Transplant rejection	D‐1 = 60u/kg, D0 = 20u/kg IP	Mouse to Rat heart re‐transplantation	Depletion of complement extends the life of the graft	13, 14
Biomaterial accommodation	2× 2U (4 μg), 12 h apart	Male SW mice were implanted with gold (mercaptoglycerol‐modified) 24 h after 2nd injection of CVF	Complement integral to inflammatory response to biomaterials	15
CDC	25 μg IV D5,9 and 12 after tumor	CB‐17 SCID and CB‐17 Beige; BJAB tumor model in athymic mice; Ramos	Complement important for the efficacy of Rituximab; Campath ‐1H	16, 17, 18, 19
Asthma—airway hyper‐responsiveness	4U IP before Ozone exposure	B6; 2ppm Ozone for 3 hours	Neutrophil infiltrate much reduced after CVF use	20
IRI	12U/mouse in 0.5 ml PBS 24 and 16 h before IRI	B6—CD59, CD55 Knockout—22 min renal pedical occlusion	IRI worse in CD55 KO mice and that was due to C3 and MAC on endothelial cells	21
CNV	4U IP 2D before laser photocoagulation and every day after	B6, C3KO	CNV did not occur in the absence of complement	22, 23
Sjogren's Syndrome	1U—2× per wk for 14 wk	NOD.B10‐H2b (spontaneous)	Complement involved in salivary dysfunction, leukocytic influx, ANA and B cell profiles—ie autoimmunity	24
Atherosclerosis	20 IU/kg daily I.P. from D‐1 (surgery)	B6 APO E3‐leiden—HF‐HCD plus vein graft surgery; carotid cuff injury model in RAG‐1 KO mice	Complement involved in vein/arterial injury—thickening (atherosclerosis)	25, 26
Tumor survival	5 μg CVF IP 28, 24 and 4 h before injection of tumor cells	Nude mice + A549 (bronchoalveolar lung carcinoma)	Complement FH v C3 important to control tumors	27
Ab infusion reactions	1 mg/kg IV 5 h before HD8 Abs	WKAH rats	Complement essential for infusion toxicity to mAb therapies	28
MS	Single IP 50 μg CVF 2 days before model	B6—MOG peptide model of EAE	Depletion of complement transiently sufficient to delay disease onset	29
abdominal aortic aneurysm (AAA)	9U per mouse 2× 4 h apart D‐1; or d1,3, and 6 after surgery	B6; elastase‐induced model of AAA	Complement, particularly C3a/C5a key in disease	30
Multidrug‐resistant enterococci	4U IP—16 h prior to infection	B6; E. faecium (strain E155)	Complement depletion severely hampers bacterial clearance, leading to peritonitis and systemic infection	31
Traumatic crush injury.	15 U CVF IP D‐1	1.5‐h hemorrhagic shock, bilateral femur fracture, and soft tissue injury, followed by 4.5‐h recovery	Complement important mediator of liver damage in the model	32
Ventilator‐induced lung injury.	250 ug/kg hCVF 1 h before experiment	B6; ventilator‐induced lung injury	Complement depletion could be therapy in this condition	33
Colon Cancer	Daily IP 500 μg/kg CVF	Female B6—MC38 (5 × 10^5^) injected subcutaneously	Complement depletion helped control cancer development	34
AMD	25 μg IP D3,5	B6, C3 KO, Sprague‐Dawley albino rats	Local production of Complement important for eye disease	35
Muscle regeneration	CVF day before cardiotoxin	B6, C3, C4, fB, C3aR and C5aR + 10 μM cardiotoxin in the tibialis anterior	C3a and the AP/AL; C3aR signalling important in muscle regeneration	36

*Notes*: AAA, abdominal aortic aneurysm; Abs, Antibodies; AMD, age‐related macular degeneration; AP/AL, Alternative Pathway and/or Amplification Loop; B6, C57Bl/6; B/c, Balb/c; CDC, Complement Dependent Cytotoxity; CVF, cobra venom factor; CNV, choroidal neovascularization; CFA, complete Freund's adjuvant; D, Day; EAE, experimental autoimmune encephalomyelitis; h, hour; HRP, Horse radish peroxidase; hCVF, Human Cobra Venom Factor; HF‐HCD, High Fat High Cholesterol Diet; IP, intraperitoneal; IV, intravenous; IRI, Ischemia reperfusion injury; KO, gene knockout; RBC, Red Blood Cell; MAC, Membrane Attack Complex; MOG, myelin oligodendrocyte glycoprotein; MS, Multiple Sclerosis; PBS, phosphate buffered saline; Wk, Week. SCID, severe combined immunodeficiency; SRBC, Sheep red blood cells. N/A, not applicable.

### 1.2 Cross species similarities/differences in the AP

**FIGURE 1:**
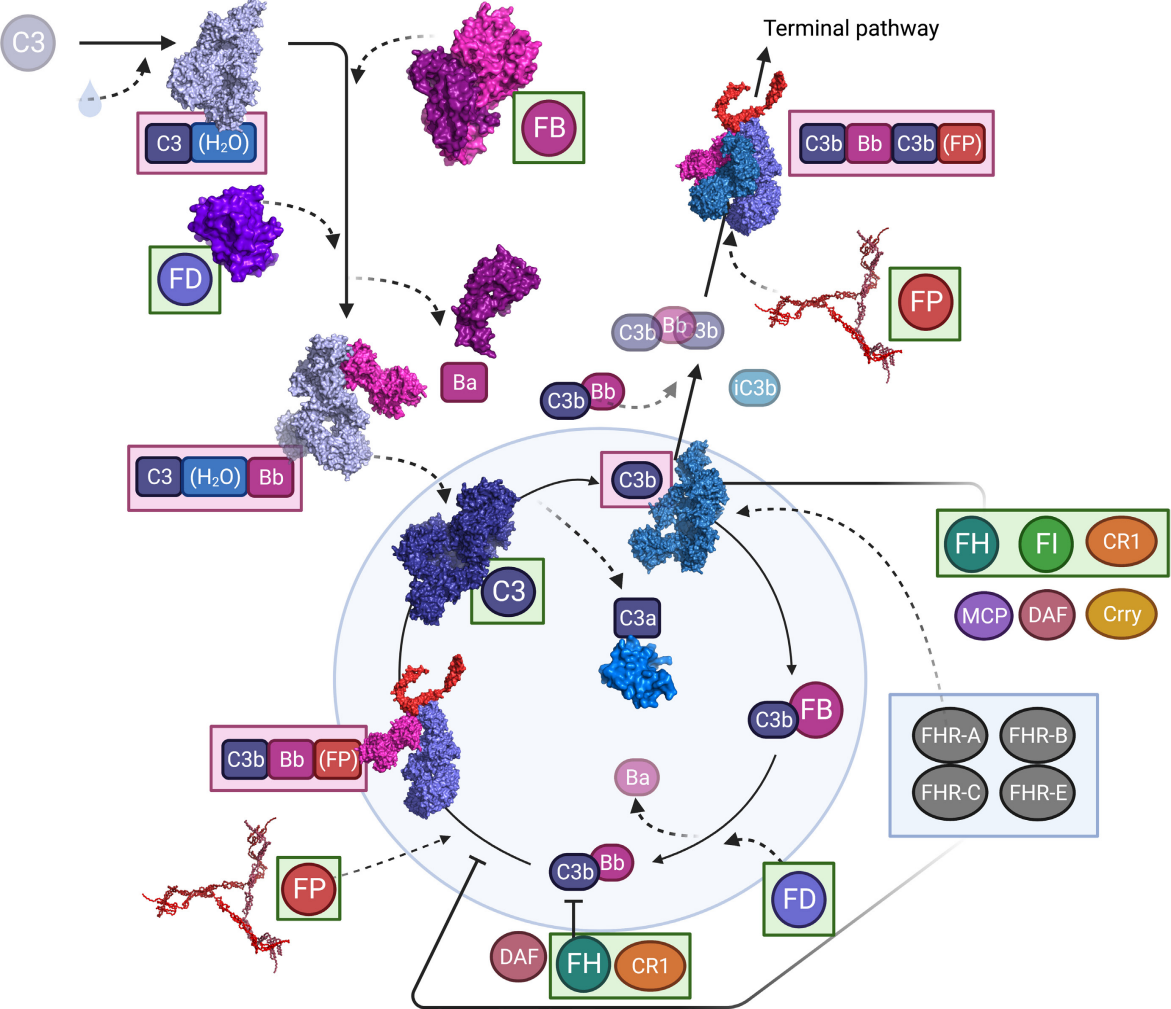
The AP/AL is highly conserved between mouse and human, enabling human proteins (green boxes) to be used and function adequately within the murine system, for example, human Factor H (FH) easily regulates C3. Conversely, human C3 is not regulated by murine FH (or Crry) but can be activated and binds to mouse Factor B (FB) and Factor D (FD) (red boxes).

Some important basics worth discussing with respect to small animal models is that the AP of mouse/rat, as in humans, includes C3, FB, FD, properdin, FH & FI, and that these proteins function in a highly analogous way (see Figure 1). For instance, the murine equivalent of FH (mFH) has been shown to be both structurally and functionally like human FH (hFH).^50^ The sequences of mFH and hFH are, overall, 61% identical. In complement control protein motifs (CCPs) 1, 2, 3 and 19, inter‐species sequence identities are between 69% and 78%; but values for key surface‐recognition CCPs, 7 and 20, are only ~50%.^51^, ^52^ Furthermore, both mouse and man also possess an array of FH‐related proteins (FHRs).^53^, ^54^ For instance, FHR‐E is likely the murine homolog of hFHR‐1.^53^, ^55^ Recent work in a cfhr1 (FHR‐E) knockout mouse suggested a protective role for this protein in response to LPS challenge, with the authors concluding that FHR‐E is potentially competing with properdin rather than FH for binding the C3 convertase—which then exacerbates AP activation and its downstream effects.^55^ To us, this suggests an area of study that still needs to be fully resolved, that is, what is the interplay of FH, the FHRs and properdin in the mouse complement system?

The role of mFH seems to reflect that of hFH;^50^ however, the ligand‐binding sites on mFH have not been fine mapped as they have on hFH. Recombinant mFH binds mC3b with *K*_D_ = 1 μM and exhibits decay accelerating activity (DAA) towards both mC3bBb and (less effectively) hC3bBb.^56^ It has also been shown that mFI acts with hFH to cleave hC3b.^47^, ^48^ Importantly, successful reversal of uncontrolled AP activation in *Cfh* ^−/−^ mice following supplementation with hFH, shows competent inter‐species functional cross‐over.^57^ Interestingly, while hFH is highly effective in the cleavage of mouse C3b in fluid phase assays, the same was not seen when mFH was used to regulate human C3b.^58^ This is completely consistent with the findings recently described in the humanized C3 mouse model of C3G,^59^ indicating unidirectional effects of inter‐species/evolutionary changes between the N terminus of FH and C3b. Furthermore, lack of appropriate regulation of human C3b by CRRY^60^ likely contributes to pathology in this model. The data from the human C3 humanized mouse does infer that human C3 and mouse FB do function together and that a viable AP C5 convertase is generated.^59^ We also know that hFD can act as a viable substitute for mFD in the hemolytic assays.^47^ Similar experiments with human sera and *GPI*‐*DAF* ^−/−^ mouse‐derived red cells confirmed that mouse DAF could regulate human complement activation (via both alternative and classical pathways).^61^ In many ways, the studies in mice with hC3 and hC5 highlight that protein‐to‐protein interactions are conserved to a greater degree within species than across species.^62^ That said, conservation of overall function across species is often found and it is fortunate for our understanding of the AP/AL that hFH works so well in mouse models, allowing analysis of many putative anti‐complement therapeutics.^58^, ^63^, ^64^, ^65^

### 1.3 C3 glomerulopathy and the *Cfh* ^−/−^ mouse: A quintessential AL/AP mouse model

C3 glomerulopathy (C3G), encompassing dense deposit disease (DDD) and C3 glomerulonephritis (C3 GN, previously membranoproliferative glomerulonephritis (MPGN)), describes rare forms of glomerulonephritis (annual incidence ~2 per million population) with common clinical features and overlapping histological appearances.^66^ FH deficiency in man^67^, ^68^ and pigs^69^ leads to almost complete C3 consumption, due to lack of control on the AL; with FH deficiency linked to several disease conditions including systemic lupus erythematosus (SLE),^70^ meningococcal disease,^70^ familial hemolytic uremic syndrome,^67^ DDD,^68^ glomerulopathy^71^ and glomerulonephritis/IgA Nephropathy^70^ as well as porcine MPGN type II.^69^ From these findings, the essential nature of FH in protecting the kidney from uncontrolled AL/AP activation was inferred, including the conservation of this role across species. Therefore, generation of a *Cfh* ^*−/−*^ mouse offered the potential of an animal model to dissect the role of FH in renal disease.

This challenge was taken up by Matthew Pickering and colleagues in Marina Botto's Laboratories, Imperial College London, using standard gene knockout technology (i.e., a 2Kb section of *Cfh* including exon 3 was replaced by the Neo^r^ gene).^72^ The resulting *Cfh* ^*−/−*^ mice were described as viable and fertile under specific pathogen free conditions. As expected from the studies in pigs and man, loss of murine FH caused almost complete C3 consumption,^67^, ^68^, ^69^, ^72^ with any detectable C3 ascribed to C3b. *Cfh* ^*+/−*^ (heterozygous) mice also had lower C3 levels, suggesting a direct correlation with FH and intact C3 levels. *Cfh* ^*−/−*^ mice (6/26) succumbed to suspected renal disease by 8 months of age, with florid C3 and C9 deposition in the glomeruli of the mice, with some evidence of IgG deposits. Thus, *Cfh* ^*−/−*^ mice appeared to present with many of the salient features of C3G,^66^ although the authors did note changes in the distribution of the C3 deposits between mouse and man, potentially due to differing levels of regulation provided by FH & Crry (mouse) versus FH, MCP & CR1 (man) in the glomeruli, respectively.^72^, ^73^ The fact that glomeruli C3 deposits preceded electron dense deposits and MPGN‐like changes suggests that the dysregulated AP precipitated the eventual renal failure.

To confirm this, the authors backcrossed the *Cfh* ^*−/−*^ mouse with a *Cfb* ^*−/−*^ mouse.^74^ The *Cfb* ^*−/−*^ mouse was developed several years earlier, again by standard gene knockout technology, and despite initial fears, this gene deletion would be embryonically lethal, mice were found to be essentially healthy.^74^ No AP function was noted in the mice, and while classical pathway‐dependent hemolytic activity was largely intact, it was lower, providing a clear indication of the importance for the AL in the lytic potential of the CP. These mice therefore provided the ideal resource to block C3 activation in the *Cfh* ^*−/−*^ mouse, and with no AP function, all systemic and renal phenotypes were corrected in the *Cfh* ^*−/−*^ *.Cfb* ^*−/−*^ (double knockout) mice, confirming the causative link between AP/AL and phenotype.

In contrast to C3b, iC3b and its metabolites cannot interact with FB or properdin to form a C3 convertase. Studies in man had established that deficiency in FI lead to loss of many complement‐mediated functions,^75^, ^76^ through consumption of C3, and these deficits were completely restored by purified protein infusion.^77^ Given this knowledge, loss of FI in the mouse was therefore expected to lead to a consumption of C3 and increased susceptibility to disease. *Cfi* ^*−/−*^ mice were generated using standard knockout technology (deleting exon 4 of the *Cfi* gene)^78^ and as with the other AP knockout mice, no gross effects were noted with respect to viability and fertility. However, as predicted, serum C3, FH, and FB levels were all significantly lower in *Cfi* ^*−/−*^ mice compared with wildtype or *Cfi* ^*+/−*^ littermates.^78^ Furthermore, the remaining C3 detected in the *Cfi* ^*−/−*^ mice was established to be C3b, suggesting full consumption of intact C3, essentially as found in man.^75^, ^76^ Despite this consumptive depletion of C3, none of the mice developed C3G by 8 months, a point where all *Cfh* ^*−/−*^ mice will develop a recognizable C3G phenotype.^72^, ^78^ Thus, the *Cfi* ^*−/−*^ mouse phenotype was arguable closer to that of the *Cfd* ^*−/−*^ mice (covered later) than the *Cfh* ^*−/−*^ mice (where C3 deposition is on the GBM), although all 3 mice exhibit recognizably different renal phenotypes in 8‐month‐old mice.^72^, ^78^, ^79^ In a significant step to our understanding C3G pathogenesis, the phenotype in the *Cfi* ^*−/−*^.*Cfh* ^*−/−*^ mice was found to be identical to the *Cfi* ^*−/−*^ mouse, demonstrating that FI was necessary for GBM mediated deposition of C3 and therefore, C3G.^78^ C3 consumption in *Cfh* ^*−/−*^ mice is essentially absolute while in the *Cfi* ^*−/−*^ mice (or *Cfi* ^*−/−*^.*Cfh* ^*−/−*^ *or Cfi* ^*−/−*^.*Cfh* ^*+/−*^), C3 levels are about 25‐30% normal. Only when *Cfh* ^*−/−*^ or *Cfi* ^*−/−*^.*Cfh* ^*−/−*^ mice have FI present are iC3b and C3c (and presumably C3d,g) fragments generated and detectable in the serum. Furthermore, C3 activation products are rapidly deposited on the GBM; within 72 hour in the case of *Cfi* ^*−/−*^.*Cfh* ^*−/−*^ mice infused with *Cfh* ^−/−^.*C3* ^−/−^ sera (replete in mouse FI but no C activity).^78^ In keeping with this, *Cfi* ^*+/−*^.*Cfh* ^*−/−*^ mice have florid C3 deposition on the GBM, underlining the necessity for FI and C3 fragments in the pathogenesis of C3G. The importance of inactivated fragments of C3b in C3G was further explored in an additional double knockout strain—the *Itgam* ^*−/−*^(encoding CD11b, part of the CR3 dimer).*Cfh* ^*−/−*^ mice.^80^ Greater renal disease, including albuminuria, cellular infiltrate, hematuria, and mortality were noted in *Itgam* ^*−/−*^.*Cfh* ^*−/−*^ mice compared with *Cfh* ^*−/−*^ mice as they aged to 8 months, particularly in animals exposed to greater risk of infection (with presumably higher immune responses and local CP activation). Bone marrow transfer experiments established that loss of CR3 on white cells drove the renal phenotype. Therefore, while unexpected, signaling via CR3 was protective in *Cfh* ^*−/−*^ mice and to our mind links back to the importance of the AL/AP in immune complex solubilization and handling as well as the importance of complement breakdown fragments generated by the AL/AP in normal physiological processes. Similar far reaching and complex effects of the AL/AP have also been noted in *Cd55* ^−/−^ (decay accelerating factor, DAF knockout) mice,^61^ which have hyperactive T_H_1 responses^81^, ^82^ while *C3* ^*−/−*^, *Cfd* ^*−/−*^, and *Cfb* ^*−/−*^ animals display significantly reduced antigen presenting cell (APC) function and associated T cell responses.^83^, ^84^, ^85^, ^86^

In the mouse, Crry fulfils the role of both hDAF and hMCP in control of C3 activation at many surfaces^60^, ^87^ and mice deficient in Crry developed using standard gene technologies (disrupting exon 5 of *Cr1l*) are embryonic lethal.^88^ Heterozygous germline mutants appeared healthy and fertile, however, on intercross, no Crry^−/−^ were recovered from 245 births. Thus, control of the AP is critical for embryonic survival beyond 9.5 days of development as Crry is the only complement regulator available to control the AP/AL at these sites. Backcross of Crry^−/−^ mice onto the C3‐deficient background rescued the phenotype and confirmed the role of complement, influx of polymorphonuclear leukocytes and inflammation in the demise of the embryo.

The *C3* ^−/−^ used by Molina and colleagues above were developed by standard knockout technology (targeting the promoter and exon 1 *C3* gene but with Neo^R^ in the reverse orientation to reduce the risk of a truncated C3 being expressed under the Neo^R^ promoter).^89^ Mice were readily generated and were healthy/viable until given an i.p. injection of S.pneumoniae. C3^−/−^ mice showed a nearly 2000‐fold increase in bacterial load compared with C3^+/−^ littermates and had much poorer survival profiles. However, these mice appeared to generate a pro‐C3 which may leave any intracellular roles for C3 intact despite complete loss of serum complement activity.^89^ Of note, these C3^−/−^ mice are distinct from the more commonly used C3 null mouse developed in the laboratories of Mike Carroll (Harvard medical school) which are available from the Jackson Laboratory, Bar harbour, Maine, US (Strain #:003641—B6;129S4‐C3^tm1Crr/J^). The C3^tm1Crr/J^ (*C3* ^−/−^) mice were also developed by standard knockout technology (where the NeoR gene replaced the sequence encoding the arginine rich linker of pro‐C3) and were found to be extensively healthy but had a markedly impaired responses to group B steptococci.^90^ As these data largely speak to the role of C3 in the immune system, we will not dwell on them, but suffice to say the C3^−/−^ mice have helped revolutionize our understanding of the role for complement in an ever‐increasing number of diseases and conditions.

Returning to the Crry deficient mouse, it was demonstrated that embryonic lethality had an absolute requirement for the AP/AL,^91^ as double FB^74^ and Crry‐deficiency resulted in normal embryo development, while mice devoid of IgG (and B cells, μMT^−/−^) and Crry failed to correct the phenotype. Further confirmation that the classical pathway played no role in the loss of embryos was noted in mice deficient for C4 and Crry, which were embryonic lethal. Surprisingly, deletion of C5 in the context of Crry‐deficiency also failed to protect embryos from AP/AL activation. Thus, the AP/AL was the only part of the complement system involved in this phenotype.^91^ Through strategic breeding between *C3* ^−/−^.*Crry* ^−/−^ males with *C3* ^+/+^.*Crry* ^+/−^ females, the authors were able to discern that it was maternal C3 that was responsible for the complement mediated loss of the embryos. It was later shown that properdin deficiency could protect *Crry* ^−/−^ mouse from AP‐mediated embryonic death.^92^

*Cfp* ^*−/−*^ mice were first developed by Cordula Stover and colleagues, using standard knockout technology (replacing exons 3‐7 with *Neo* ^*r*^) and deleting the thrombospondin repeats (TSR) with importance for binding to C3b.^93^ As predicted, rabbit red blood cell (RRBC) lysis by *Cfp* ^*−/−*^ sera (in the presence of EGTA) was significantly less than noted in wildtype mice. Using a sepsis model, it was evident that properdin was critical to animal survival and that properdin may interact directly with bacteria to trigger and sustain complement activation. These data fit well with our understanding of properdin as a potential initiator and as the principal positive regulator of the AP/AL. Based on these data, loss of positive regulation of the AP/AL through loss of properdin was predicted to ameliorate C3G but that was not the outcome.^94^ *Cfp* ^*−/−*^.*Cfh* ^−/−^ mice had very little detectable C3 in their plasma, that is, not significantly different to *Cfh* ^−/−^ mice unlike the essentially normal levels in the *Cfp* ^*−/−*^ mice. However, C5 plasma levels were strikingly different between *Cfp* ^*−/−*^.*Cfh* ^−/−^ (readily detectable) and *Cfh* ^−/−^ (almost undetectable) mice, suggesting an important role for properdin in C5 consumption/activation in *Cfh* ^−/−^ mice.

In experiments that paralleled those above and an attempt to develop a mouse model of aHUS, Song's group used gene knock‐in technology to insert a two stop codons at the beginning of CCP19 of mouse FH.^95^ Viable mice were generated (termed FH^m/m^) but only a small amount of the mutated FH was detectable in the mouse plasma (presumed a result of an unstable FH mRNA product), insufficient to prevent uncontrolled consumption of C3 and FB. These mice had a renal phenotype akin to the *Cfh* ^−/−^ mice, which again, over time (12 months), developed into a recognizable C3GN. The authors termed this model an “age‐dependent C3 GN”.^95^ Next, the authors backcrossed to an additional properdin deficient line (exon 5 and 6 swapped for NeoR with intended flanking LoxP sites to generate a conditional knockout mouse) but due to a suspected unusual homologous recombination event the 5 prime loxP site was not transferred to the selected animals and this led to loss of expression of properdin mRNA/protein.^96^ Experiments with this properdin deficient mouse demonstrated that properdin is critical for lipooligosaccharide and lipopolysaccharide (LPS) mediated activation of the AP. Their experiments also confirmed that human properdin can substitute for mouse properdin in certain contexts in the activation of the AP in mouse serum, linked to how well human properdin interacted with different bacterial preparations. They also showed that properdin was critical for activation of the AP on red cells from *Crry* ^−/−^ mice but zymosan mediated AP activation was not significantly disrupted by the lack of properdin. Importantly, these in vivo studies demonstrated that properdin was important for context dependent AP activation rather than necessary for sustained activation of the AL. When this *Cfp* ^−/−^ mouse was backcrossed to the FH^m/m^ mice, as with the previous study,^94^ animals developed an aggravated renal phenotype (when compared to FH^m/m^ or *Cfp* ^−/−^ mice alone) with all mice dying as a result of renal disease between 8 and 12 weeks.^95^ The renal disease being described as similar to DDD and independently corroborated the findings from Imperial,^94^ albeit not precisely. Other than renal disease and its associated pathologies, a loss of fat cells in the peripancreatic fat pad was noted, this will be discussed later.^95^ Furthermore, these data confirm properdin can provide positive effects, its loss (or low levels) also being found to unexpectedly lead to increased atherosclerotic plaque progression^97^ and to increased severity of heart failure in man.^98^

The Imperial studies also confirmed differential staining patterns in the kidney in the presence and absence of properdin, suggesting that properdin was facilitating consumption of complement at particular surfaces in the kidney, again demonstrating that properdin is more important in activation of the AP at particular sites than it is in sustaining the AL in the fluid phase.^94^, ^95^ These data indicated that in the absence of FH (and FI) regulation, the availability of additional intact C3, as a result of less activation driven at surfaces, and consequently more intact C3 being available in the fluid phase, was actually detrimental and that this scenario must be avoided in treatment of renal diseases.^94^, ^95^ These studies also raised the concept of a Rubicon event in the control of C3 activation, that sufficient fluid phase regulation via FH and FI is required to stabilize plasma C3 levels and that even low levels of FH is sufficient to alter outcome. This scenario was further explored in a publication where FH was deleted in hepatocytes using standard conditional gene deletion approaches.^99^ The *cfh* ^loxp/loxp^ mice (loxP sites engineered 3 prime exon 2 and 5 prime exon 3) had reduced FH plasma levels, suggesting an impact on the expression of this gene across the mouse. This is reminiscent of, although not as marked as, the issues encountered in the generation of the properdin deficient line developed in Song's group above.^96^ However, on backcross onto the hepatocyte (Albumin promoter) Cre recombinase expressing mice there was a complete loss of FH production from hepatocytes and serum FH levels of were found to be 19% (median) of wildtype levels.^99^ Animals with reduced FH had evidence of increased C3 deposition but renal disease was mild by comparison to *Cfh* ^−/−^ and *Cfh* ^−/−^.*Cfp* ^−/−^ mice. The data again confirmed the need for FH to protect the GBM and indicated that even 20% normal levels was sufficient to protect this particular surface. Another interesting mechanistic insight from this study is that even low levels of FH can restrict AP C5 convertase formation and the consumption of C5. The data also highlighted the role of C5 activation in the formation of thrombotic microangiopathy (TMA) in the kidney and provided a model system that allows analysis of aspects of C3G and aHUS, a phenotype that can be seen in the clinic.^99^ In summary, soluble complement regulator FH and its role in controlling the AP/AL is critical to prevent the development of complement mediated kidney disease in humans, pigs, and mice.^67^, ^69^, ^72^ Furthermore, FH is crucial for the regulation of complement activation on the extracellular matrix,^100^ that is, GBM and sits in opposition to activation of the AP via properdin (and further modulated by the FHRs). In the absence of FH, spontaneous activation of the AP occurs leading to complete consumption of C3 and FB,^101^ and in this manner, FH deficiency is highly analogous to use of CVF to actively deplete complement. Consumption of C3 and FB are frequent in C3G,^102^ but complete FH deficiency is an extremely rare event, functional deficits are more readily observed and these can manifest as either C3G or aHUS underscoring the role of modifiers in the outcomes.

The FH‐related proteins (and properdin) are such modifiers^103^ and with this understanding the Pickering group embarked on an ambitious project to convert the mouse FH locus to that of the human.^104^ Their first step was a CRISPR/Cas‐mediated gene edit to generate mice devoid of the entire 664 kb FH‐FHR (delFH‐FHR) or the 537 kb FHR locus (delFHR). As the original *Cfh* ^*−/−*^ mice, animals devoid of FH and their FHRs had very little intact C3 in circulation while deletion of all FHRs had no apparent impact of C3 levels. Renal phenotype in 8‐month‐old mice was also established to be comparable between delFH‐FHR and *Cfh* ^−/−^ mice.^72^ With the transgenic introduction of both human (h)FH and hFHR5 (the mutant version associated with CFHR5 nephropathy^105^, ^106^) or the *hCFH* and native *hCFHR5* genes to the delFH‐FHR strain, partial humanization of the mouse FH locus was achieved. These mice had differential expression of hFH allowing the authors to note the direct correlation with hFH level and that of C3/C5 in the transgenic delFH‐FHR mice. Only hFH‐FHRmut (delFH‐FHR) mice demonstrated pathogenic deposition of C3, properdin and FH/FHRmut protein in the kidney.^104^ Next, compelling evidence that FHR5mut protein possesses a dominant negative effect (in terms the gain‐of‐function mutant precipitating disease) in the renal environment was shown through analysis of mice expressing both FHR5 and FHR5mut proteins. The effect being put down to the ability of the protein to outcompete hFH for binding to C3b in the glomerulus. Using our homodimeric minimal FH construct^65^ in a gene therapy format, the authors demonstrated that the superior binding affinity of FH (when in a dimer conformation) was sufficient to gain access to and control the AP convertase—clearly showing the importance of this functionality in certain disease scenarios. These data again suggest that in certain circumstances, particularly in the case of mutant FHR proteins, the FHRs can alter the balance of regulation of deposited C3b and interfere with FH/FI‐mediated breakdown. Our understanding of the full in vivo physiological importance of the interactions of FHRs, properdin and the regulators of the AP/AL C3 convertase in kidney (and in other organs) is still in its infancy. We suspect these particular humanized models, and no doubt other humanized mice of this sort that are likely to follow, will provide key important understanding of normal processes, disease mechanisms and reveal drug targeting opportunities.

Humanizing the mouse complement system is now becoming common place due to the advances in gene manipulation technologies but requires careful consideration. One early example was mice humanized with full‐length hFH, which was successful in reversing kidney disease in transgenic mice with expression levels closer to normal mouse FH expression (denoted *CFH‐H*, see later) but not in mice with low hFH (*CFH‐Y*) expression.^107^ These data fit well with the mice generated by Vernon et al.^99^ We had considered this approach (using hC3) when developing the C3 GOF mouse model of aHUS (see later), but we ruled it out due to the unknowns surrounding the ability of mouse complement receptors and regulators to interact in an expected way with the human protein. These concerns were borne out in a recent study where a humanized C3 mouse was developed using Velocigene technology.^59^ ^,^ ^108^ The knock‐in (knockout) approach was confirmed as mice showed no expression of mouse C3 but low expression of hC3 (C3 slow variant), roughly 33% at the transcript level and only 4.5% in serum. Mouse C5 levels were reduced, and mice exhibited spontaneous mortality, median survival of 16 weeks.^59^ The infusion of serum purified hFH (but not hFI) into the mice was able to increase hC3 plasma levels, suggesting a need for hFH to regulate hC3 in these mice. Our own studies confirmed that hFH does not regulate mC3 in fluid phase assays,^58^ confirming the authors' assertion that this is likely the main reason for the mouse phenotype. Again, the importance of the interaction between FH and C3b in the kidney is demonstrated.^59^ The humanized C3 mouse now represents a model of C3G based on a C3 gain‐of‐function, that is, that key binding interactions with mFH have been lost, we would speculate these are in the N‐terminal domain given the phenotype. In the model, they used an in‐house generated anti‐C5 mAb and demonstrated only partial protection. The findings again show an important role for the terminal pathway in renal disease but also demonstrate that C5 independent and AP/AL factors remain integral to pathogenesis and end‐stage renal failure in the mice.^59^ Indeed, use of agents that blocked the AP (anti‐C3b or anti‐FB) had a marked impact on animal survival while C2‐deficient hC3 and hC3/mC3 (heterozygous) mice had a survival profile identical homozygous hC3 mice—that is, the uncontrolled hC3 overwhelms any regulation of endogenous mouse C3 and the CP and LP are not the main drivers of disease.^59^

### 1.4 Atypical hemolytic uremic syndrome (aHUS): A prototypical AP‐mediated disease

Hemolytic uremic syndrome (HUS) is defined as the clinical triad of microangiopathic anemia, thrombocytopenia, and acute renal injury. This can be sub divided into Shiga toxin‐associated HUS (STEC‐HUS) and non‐diarrheal or atypical HUS (aHUS). STEC‐HUS is the most common form, accounting for >90% of cases and is linked to preceding illness with Shiga toxin‐producing bacteria.^109^ Atypical HUS is rare (incidence 0.53/million population^110^) and can be further categorized as sporadic or familial aHUS. Susceptibility to the disease is multi‐factorial, illustrated by ∼50% penetrance of the phenotype. The complement system, particularly FH, was shown by the Newcastle complement group to be central to the pathogenesis of aHUS in 1998^111^ and since then, multiple other AP components have been associated with the disease. Indeed, in the most comprehensive analysis to date, >600 rare variants in 10 complement genes have been identified in over 3000 aHUS patients (identified in 6 reference centers including our own).^112^ Thus, aHUS arguably represents an archetypal complement mediated disease.

The group at Imperial College London were the first to describe a mouse model of aHUS based on the appreciation that aHUS‐associated defects were predominately in the C terminus of Factor H and distant from the complement regulatory domain in the N terminus (where mutations were more commonly associated with C3G and AMD).^113^ They therefore developed a transgenic mouse which expressed a truncated mouse FH protein (FH CCPs 1‐15) designated the FHΔ16‐20 mouse. This transgenic mouse was then backcrossed to the *Cfh* ^−/−^ mouse to generate a mouse that only expressed the truncated FH protein.^113^ The mice were fully viable, and the truncated protein expressed at level equivalent to *Cfh* ^+/−^ mice, sufficient to prevent accumulation of C3 on the GBM (as noted above for *Cfh* ^−/−^ mice). The *Cfh* ^−/−^.FHΔ16‐20 mice rapidly succumbed to renal disease characterized by hematuria and TMA, and no evidence of electron dense deposits by EM. In short, the development of this mouse demonstrated that the carboxy terminus of FH is essential to prevent aHUS‐like disease.^113^ Furthermore, the study also demonstrated, using a mouse with low level (2% of FH levels) expression of the FHΔ16‐20 protein, that fluid phase AP control was needed to leave enough intact C3 available to drive membrane/surface targeted damage. This mouse did not develop C3G and again the study demonstrated that a low concentration of FH was sufficient to regulate fluid phase amplification and prevent murine disease. The finding that *Cfh* ^+/−^.FHΔ16‐20 mice did not develop any disease (unlike patients who routinely display one normal and one mutant allele) also provided support to the concept that additional modifiers were important to precipitate disease in aHUS patients and by extension, that functional deficits in the AP alone were not the full story in aHUS. The *Cfh* ^−/−^.FHΔ16‐20 mouse is no longer available but additional approaches to explore the AP in aHUS were developed. This includes a mouse developed with a single point mutation in the C terminus of mouse FH (i.e., FH W1206R) based on a mutation (FH W1183R) identified in aHUS patients.^114^, ^115^ Using standard knock‐in technology (essentially as with the FH^m/m^ mouse^95^), the single amino acid change was successfully introduced with no effect on protein production, indeed if anything levels of the mutant protein were slightly elevated in the plasma of the FH^R/R^ mice (as they were denoted).^114^ However, this change led to significant consumption of C3, FB, and C5 and loss of AP activity. That said, there was still sufficient lytic activity in the serum to lyse “PNH‐like (*Crry* ^−/−^.*DAF* ^−/−^.*C3* ^−/−^; see below)” red cells. FH^R/R^ mice failed to thrive (30% died within 30 weeks postpartum) and had several “clinical” or pathological features consistent with those seen with aHUS patients.^114^ However, thrombi were detected widely in FH^R/R^ mice, with large clot formation in many organs, particularly the liver and brain and about a third of the FH^R/R^ mice developed neurological abnormalities consistent with thrombotic and hemorrhagic stroke,^114^ conditions not routinely seen in aHUS patients. Therefore, findings in this model must be reviewed in that context. Interestingly, while genetic deletion of properdin completely reversed disease in FH^R/R^ mice, it did not reduce C3 and C9 loading in the kidney.^115^ This again highlights the importance of fully functional FH in the renal environment in balancing AP/AL activity. Depletion of properdin using mAb 14E1 confirmed that a relatively short‐term reduction in properdin levels also ameliorated disease in this model. Using similar approaches, Hourcade and colleagues successfully demonstrated the ability of both rabbit polyclonal and hamster monoclonal antibodies (i.e., H4) against a recombinant properdin domain to protect animals from elastase‐induced abdominal aortic aneurysm.^116^ This model is also complement‐dependent and complement depletion blocks aneurysm formation.^30^, ^117^ These data fit with several studies demonstrating the effectiveness of properdin blockade or deficiency in ameliorating inflammatory conditions,^92^, ^115^, ^117^, ^118^ highlighting the importance of the AP/AL in these situations.

Developed in parallel to the FH^R/R^ mouse, (we) Kate‐smith Jackson et al. focused on C3 mutants associated with aHUS in a large patient cohort. After selecting and testing several patient‐associated mutants using in vitro cell culture analysis, we focused on a C3 D1115N mutation, found in three families world‐wide.^119^ This change resides in the thioester domain of C3 and at the interface of the interaction with C3 and the C terminus of FH. The D1115N change was denoted a C3 gain‐of‐function (GOF) mutation, in that FH binding was significantly reduced due to a predominant loss of C terminal binding, while N‐ terminal interactions with mFH were largely maintained. C3^D1115N^ also interacted appropriately with Crry. Homozygote mice (for consistency we will adopt the Song nomenclature approach, thus C3^N/N^) display all the hallmarks of aHUS, that is, acute kidney injury (elevated BUN, proteinuria, and hematuria), thrombocytopenia, anemia and thrombotic microangiopathy (evidenced in renal histology) associated with significant AP dysregulation (systemic changes in C3, C5 turnover as well as significant glomerular C3 deposits in the glomerulus). This phenotype can be ameliorated by anti‐C5 mAb, replicating human disease.^119^ C3^N/N^ mice (up to 6 months of age) did not have evidence of extra‐renal disease (unlike the FH^R/R^ mice described above), and therefore, appear more faithful to aHUS in man. We also have begun to tease apart the molecular pathways that drive disease via backcrossing our model onto properdin, C7 and C5aR deficient mice to aid understanding and drug target design. While clearly distinct, our findings share some similarities with those noted for the FH^R/R^ mouse. For instance and most relevant herein, we found properdin deficiency was protective on C3^N/N^ mouse background. Over 6 months, only 2 of 13 C3^N/N^.*Cfp* ^−/−^ mice succumbed to disease compared with 14 of 15 C3^N/N^ mice. C3^N/N^.*Cfp* ^−/−^ mice had no evidence of hematuria, proteinuria, or thrombotic microangiopathy (TMA, analysis on going). There was no evidence of a dose‐response as C3^N/N^.*Cfp* ^+/−^ were not protected and succumbed to disease in a similar fashion to properdin sufficient C3^N/N^ (Kamala et al, in progress). During this study, we also note that C3^N/N^ have low serum levels but significant renal properdin deposits in comparison with wildtype controls. These data potentially indicate that consumption of properdin is a factor in disease progression or that, as with other models, where high kidney C3 deposits are noted, that properdin is sequestered. This makes some sense in that the lack of FH binding to C3 convertase in the model should allow more properdin to bind and remain resident. Properdin deficiency did not appear to alter C3 deposit load in the kidney, again mirroring the FH^R/R^ results, suggesting C3 convertase remains highly active generating significant deposits of C3b. Therefore, given the success of anti‐properdin or properdin deficiency in ameliorating disease in both the C3^N/N^ and FH^R/R^ models of aHUS, where both have defined TP dependency, it seems likely that these effects are related to loss of stability in the AP C5 convertase.^89^

### 1.5 Insight from “transplant” models

The development of DAF (CD55) deficient mice^61^ afforded analysis of the role of complement in other phenomena including ischemia reperfusion injury (IRI). Here, *DAF* ^−/−^ mice had much poorer renal function during a bilateral renal IRI model than WT (or CD59a^−/−^); Double *DAF/CD59* ^−/−^ compounded this effect significantly. More severe tubular damage and neutrophil infiltrate was noted in DAF deficient mice, which again was further amplified in the double knockout mouse. Depletion of C3, by CVF, prior to IRI induction in the DAF/CD59 double knockout mice confirmed a role for complement in the renal damage. Furthermore, detailed analysis of C3 deposits confirmed that C3 deposits are routinely seen on tubular cells in healthy mice but that C3 (and C9) deposition in the peritubular capillaries indicated that MAC‐induced microvascular injury was the principal pathogenic mechanism for complement in this model of IRI. Zhou et al illustrated that mice deficient in C3 are protected from IRI and that was not seen in C4 deficient mice.^120^ Additionally, Ig deposition (IgM and IgG) was absent in a renal IRI model while complement deposition was still present in RAG‐1^−/−^ mice^121^— all of which suggest the AP/AL plays a predominant role in IRI in the mouse kidney. In definitive works, which also further underlined the utility of the *Cfb* ^−/−^ mouse to tease apart the role of the AP/AL in pathology, Thurman et al demonstrated that loss of FB significantly protected the kidney from reperfusion injury.^122^ These data echoed those from an early study in the MRL/lpr disease model (immune complex nephritis), where loss of FB was protective, in that mice had significantly less renal disease, immune complexes and a normalized C3 level.^123^ In experiments designed to elaborate on the role of complement in driving the wider inflammatory consequences of IRI, FB deficient and sufficient mice were subjected to IRI.^124^ Gene array analysis revealed that expression of a number of chemokines were linked to complement activation. Subsequent experiments demonstrated that C3a and not C5a was critical in the generation of the response and this was mediated via the NF‐kB pathway.^124^

While all tissues are not equal, FB deficiency is protective in a model of transient coronary ligation.^125^ Furthermore, deficiency of FB (either genetic or via mAb therapy) is integral to the hyper‐responsiveness and inflammation in both ovalbumin and ragweed sensitized airway allergy models.^126^ These studies followed work showing the importance of C3a and C3aR in the disease model^127^, ^128^ and highlight the importance of the AP/AL to the immune response as well as intrinsic tissue damage. Elegant (and frankly heroic) studies using triple genetically modified mice (DAF/CD59 and properdin or FB) or anti‐properdin monoclonal antibodies were used to show the importance of properdin and the AP/AL in IRI as well as confirm the importance of C3aR and C5aR mediated inflammation.^118^

In a mouse stroke model, it was established that *Cfb* ^−/−^ mice and those treated with CR2‐FH (an AP inhibitor,^129^, ^130^ see Table 2) were significantly protected from the negative effects associated with this cerebral IRI.^141^ This study was extended to look at total complement inhibition versus AP/AL inhibition, interesting while total C3 deficiency was initially protective, AP/AL inhibition had the best overall protective effects, due to C3 deficiency leading to greater risk of postoperative infection, which were controlled by antibiotics.^145^ Furthermore, the authors indicated that complement activation while suppressed in the presence of CR2‐FH was still adequate to activate the lectin and classical pathways, stimulating recovery and neurogenesis during the early stages of injury.^145^ These studies link with other neurological studies in mice that suggest that total ablation of C3, and presumably the AP/AL, can be a deficit. As noted above, a level of tonic C3 function is important in the brain, eye and of course the immune system. In short, these studies all highlight the importance of the AP/AL in the pathogenesis associated with IRI but also remind us that AP/AL homeostatic functions are also incredibly important.

**TABLE 2:** Use of CR2‐FH (or TT30) in animal models: illustrating the integral role of AP/AL in disease mechanism

Year	Target disease	Model	Dosing strategy	Reference
2008	IRI	B/c; Intestinal—40 min ischemia, 120 min reperfusion	CR2‐FH—Protein infusion; I.V. 30 min after reperfusion—range of doses 0.025 up to 0.4 mg	130
2009	Wet AMD	B6; Laser‐induced Choroidal Neovascularization (argon laser photocoagulation—ruptures Bruch's membrane)	CR2‐FH—Protein infusion; I.V.—0.25 mg, 0, 24 and 48 h after injury	131, 132
2009	Arthritis	B6; Collagen Ab‐induced arthritis	CR2‐FH—Protein infusion; I.P.—0.25 or 0.5 mg—15 min after mAb day 0 and after LPS day 3	133
2010	IRI	B6; heterotopic isograft heart transplant into abdomen. Cold ischemia was 35 min, 30 min anastomosis—hearts were examined 12 and 24 h later	CR2‐FH—Protein infusion; I.P.—0.4 mg—immediately after surgery	134
2011	Hemorrhage (hemorrhagic shock)	B6, IL‐12p40^−/−^ and IL‐12p35^−/−^. 25% v by wt blood removal via retro‐orbital sinus	CR2‐FH—Protein infusion; I.V.—17.5 uM (estimate 0.06 mg) given 5 min after bleed	135
2011	Paraquat (PQ) Poisoning	B/c; PQ (20 mg/kg) via I.P. injection	CR2‐FH—Protein infusion; I.P.—0.8 mg—immediately after PQ injection	136
2011	Systemic lupus erythematosus	Female MRL/lpr (spontaneous)	CR2‐FH—Protein infusion; I.P.—0.4 mg—twice per week—wk 15 to 23	137
2011	Systemic lupus erythematosus (Lupus nephritis)	Female NZB/W F1 mice (spontaneous)	CR2‐FH—Protein infusion; I.P.—0.4 mg—twice per week (after onset of renal disease, proteinuria >0.1 mg)—wk 23 to 31	138
2011	Fulminant hepatic failure	B6; I.P injection of 2.5 μg/kg LPS and 300 mg/kg D‐GalN	CR2‐FH—Protein Infusion; I.V.—40 mg/kg, immediately after the LPS/D‐GalN injection	139
2012	Wet AMD	B6; Laser‐induced Choroidal Neovascularization (argon laser photocoagulation—ruptures Bruch's membrane)	TT30—Protein infusion; I.V.—250 ug, 0, 24 and 48 h after injury	140
2012	Stroke—ischemic brain injury	Male B6; Middle cerebral artery occlusion for 60 min followed by 24, 72 and 168 h reperfusion	CR2‐FH—Protein infusion; I.V.—0.4 mg at 30 min post reperfusion	141
2012	Multiple Sclerosis	B6; experimental autoimmune encephalomyelitis—D‐1 = 250 ng of PT (I.P), D0 = CFA emulsion of 1 mg heat‐ inactivated mTB and 250 μg MOG peptide35‐55; D1—repeat PT injection	CR2‐FH—Protein infusion; I.V.—0.4 mg d7,9,11,13 post induction	142
2014	Adenovirus immuno‐reactivity	B6; 10^10^ virus I.V.—200 μl PBS—HAdv5 (Ad5L), Ad5/35S, and Ad‐ts1 HAd2	CR2‐FH—Protein infusion; I.V.—0.05 mg—1 h prior to Ad injection	143
2015	Inflammatory bowel disease	B6; 4 cycles of oral administration of 3% (w/v) DSS for 7 days followed by normal drinking water for 10 days	CR2‐FH—Protein infusion; I.P.—0.25 mg given D1 during cycles 2‐4, and then every 48 h thereafter for the duration of DSS treatment.	144
2015	Stroke—ischemic brain injury	Male B6; Middle cerebral artery occlusion for 60 min followed by 24 or 168 h reperfusion	CR2‐FH—Protein infusion; I.V.—0.4 mg at 30 min post reperfusion	145
2016	C3G	FH deficient—spontaneous	CR2‐FH—Protein infusion I.V.—40 mg/Kg—single bolus	64
2016	Severe traumatic brain injury	Closed head injury (2.5 cm weight drop)	CR2‐FH—Protein infusion I.V.—500 ug; 1, 4 and 24 h following injury	146
2016	IRI; Delayed graft function	Lewis Rat—orthotopic kidney transplantation	TT30—Protein infusion—130 ug per kidney; systemic 32 mg/kg IV daily for 7 days or implanted pump (40 mg/kg/24 h, day −2 to day +7)	147
2016	AMD	Passive smoke inhalation for 6 months	CR2‐FH—Protein infusion I.V. 10 mg/Kg given 3× per week for 3 months	148
2018	Wet AMD	B6; Laser‐induced Choroidal Neovascularization (argon laser photocoagulation—ruptures Bruch's membrane)	AAV5‐VMD2‐CR2‐FH; Sub‐retinal injection—3 × 10^11^ vg/ml	149
2018	Traumatic brain injury	B6; controlled cortical impact—contusions made using pneumatic impactor device	CR2‐FH—Protein infusion I.V. 16 mg/Kg, single bolus 1 h following cortical injury	150
2018	Wet AMD	B6; Laser‐induced Choroidal Neovascularization (argon laser photocoagulation—ruptures Bruch's membrane)	CR2‐FH—Stably expressing ARPE‐19 cell encapsulated (1 × 10^6^ cells) in Alginate; ~10 capsules per eye; Intravitreal injection	151
2018	Wet AMD	B6; Laser‐induced Choroidal Neovascularization (argon laser photocoagulation—ruptures Bruch's membrane)	CR2‐FH—Protein infusion; I.P.—10 ug/g given D6 after lesion generated every 48 h until D22	152
2019	aHUS	C3D1115N GOF model—spontaneous (therapy)	CR2‐FH—Protein infusion I.P.—40 mg/kg every 24 h partially effective	153
2020	Wet AMD	B6; Laser‐induced Choroidal Neovascularization (argon laser photocoagulation—ruptures Bruch's membrane)	CR2‐FH—Stably expressing ARPE‐19 cell encapsulated (1 × 10^6^ cells) in Alginate; Subcutaneous in Matrigel 4 weeks prior to CNV	154
2021	AMD	B6: Passive smoke inhalation for 6 months (5 h a day, 5 days per wk)	AAV5‐VMD2‐CR2‐fH, AAV2YF‐smCBA‐CR2‐fH; Sub‐retinal—3 × 10^11^ vg/ml—1 month prior to exposure to smoke	155

*Notes*: AAV, adeno‐associated virus; Ab, Antibody; Adv, adenovirus; AMD, age‐related macular degeneration; ARPE, a retinal pigment epithelial cell; B6, C57Bl/6; B/c, Balb/c; C3G, C3 glomerulopathy; CR2‐FH—4 N‐terminal SCRs of mouse complement receptor 2 (residues 1‐257 of mature protein; GenBank accession number M35684) linked (natural linker amino acids KIEL) to the five N‐terminal SCRs of mouse factor H (residues 1‐303 of mature protein; GenBank accession number NM009888); CFA, complete Freund's adjuvant; D, Day; D‐GalN, D‐galactosamine; GOF, gain‐of‐function; h, hour; I.P. intraperitoneal; I.V. intravenous; IRI, Ischemia reperfusion injury; TT30, the human version of CR2‐FH. LPS, lipopolysaccharide; mAB, monoclonal antibody; mTB, mycobacterium tuberculosis; MOG, myelin oligodendrocyte glycoprotein; PT, pertussis toxin; PQ, paraquat; Wk, Week.

### 1.6 The multifarious AP/AL: Broadening the scope of Complement research

The role of the AP/AL as an immune surveillance system is well established and longstanding. However, animal models and complement antagonists which were originally created to investigate innate immunity are also providing insights into other cellular processes; for instance, *C3* ^−/−^ mice are protected against bone loss in an ovariectomized osteoporosis model,^156^ injury‐induced neurogenesis,^157^, ^158^ pregnancy‐associated complications,^159^ and inflammatory bowel disease.^160^ It is beyond the scope of this review to discuss all these scientific discoveries in detail, so we will concentrate on a few key areas: the controversy of “intracellular complement,” metabolism and metabolic disorders and age‐related macular degeneration (AMD).

### 1.7 The “complosome”: Intracellular complement: part of the AP or something different?

Local complement production by cells such as hepatocytes, adipocytes, and chondrocytes is well characterized, but in recent years, the hypothesis of intracellular complement has developed and become controversial. This phenomenon was first observed in human T cells,^161^ where intracellular cathepsin L‐mediated C3 cleavage was shown to stimulate the C3a‐C3aR signaling pathway, promoting resting and activated T cell survival. Liszewski et al. found that myeloid, endothelial, and epithelial cells also retained a store of C3 and demonstrated intracellular C3 cleavage, suggesting that the original T cell data may be applicable to all cell types.^161^ Further data to back up this assertion was provided by a murine ischemia reperfusion injury model, using *C3* ^−/−^ and wildtype hearts in isolation from systemic effects.^162^ These data suggest a protective role of C3 by modulating mitochondrial respiration and the levels of NADH and NAD+ in the heart. The authors noted an impaired redox state in the *C3* ^−/−^ hearts compared with WT, with *C3* ^−/−^ hearts also displaying significantly lower ATP levels after ischemia compared with WT.^162^ Further data from an intestinal ischemic model suggests cathepsin B can cleave newly generated/intracellular C3 in the mouse as cathepsin inhibition and cathepsin B deficient mice were protected from ischemic injury and had less deposition of C3 fragments.^163^ In addition, recent and comprehensive data using monosodium urate crystal (MSU) and Zymosan‐induced monoarthritis arguably provides data for similar roles for C3 in driving a disease state.^164^ C3 is required to drive maximal tissue priming, driving changes in synovial fibroblasts (SF) leading to a significant additional damage (irrespective of the adaptive immune system). Bulk RNA‐seq data gathered from Thy1+ SF highlighted upregulation of FB and properdin as well as the C3a and C5a receptors, in conjunction with metabolic changes. Using Confocal analysis, they confirmed increased C3 expression in primed SF, with large amounts being found in ER and golgi apparatus, and C3 being found in stores. The authors compared this to the findings in human pancreatic cells, and therefore, inferred intracellular function. The role of terminal pathway, hematopoietic cells and bystander production of C3 were all ruled out in the process of SF priming.^164^ C3 expression by SF modulated a switch of metabolic function, where C3‐deficient SF were driven into senescence, and potentially became immunoregulatory, while WT SF became activated/primed and more likely to drive disease. Additional analysis provided further links to the studies in man demonstrating the role of the mTOR and the inflammasome (*NLRP3*, in particular)^165^, ^166^ and use of C3aR deficient animals confirmed a role for the C3a‐C3aR axis in tissue priming, with a skew towards aerobic glycolysis, in the MSU model.^164^ Analysis of human scRNA‐seq data from human synovial tissue, correlates across species were established, supporting the thesis that C3 and components of the AP were important in disease progression. These data suggest that C3 inhibitors—potentially gene therapy‐based inhibitors—would be particularly interesting to evaluate in these models and potentially in clinical trials. The data may also explain why targeting the terminal pathway did not provide the benefits previously envisioned. The existence of an intracellular AP/AL remains controversial (an “alternative” alternative pathway, if you like) and studies in the mouse have not, as yet, provided sufficient clarity on the topic, most data in mice supports a role for C3 and the AP, and a role for the cathepsins in cleavage of C3 seems plausible. What is clear is that a concerted effort from the community is required to tease apart the various roles of the complement system in cellular function.

### 1.8 Systemic metabolism and metabolic dysfunction

The association of complement with the field of metabolism is also longstanding; adipose tissue produces a number of complement proteins including C3 and FB^167^ and FD. Indeed, FD is alternatively known as the serine protease adipsin due to its abundant synthesis by adipocytes.^168^ FD expression (at the mRNA level) was almost completely absent in certain acquired and genetic rodent models of obesity^167^, ^169^ indicating that obesity may prime for conditions which restrict FD expression and initially suggested a central role for FD in systemic metabolic control. However, *Cfd* ^−/−^ mice (standard knockout technology, disrupting Cfd exon 3) were healthy and viable with no apparent abnormality noted up to 26 weeks of age (similar to *Cfb* ^−/−^ mice). *Cfd* ^−/−^ animals (fed normal chow) showed no difference in body weight or serum concentrations of triglycerides, cholesterol, and free fatty acids.^47^ Of course, *Cfd* ^*−/−*^ mice displayed no AP activity in RRBC‐based hemolytic or zymosan C3 deposition assays, despite significantly increased C3 levels compared with wildtype littermates, demonstrating that FD was essential for C3H_2_OBb formation in mice. Additionally, these experiments underlined the importance of the AL/AP in opsonization of bacteria, with around 66% less opsonization seen in FD deficient sera.^49^ A novel FD deficient mouse created using the CRISPR/Cas9 system also indicated no significant difference in the body weight, epididymal adipose tissue weight or plasma glucose and insulin levels between *Cfd* ^−/−^ and wildtype animals, when fed a high‐fat diet (HFD).^170^ However, researchers noted that hepatic FD was elevated in wildtype animals on a HFD compared with those on normal chow. *Cfd* ^*−/−*^ HFD livers showed decreased hepatic lipid accumulation (independent of HFD‐induced insulin resistance) and reduced expression of genes involved in fatty acid synthesis and the PPARγ/CD36/FATP2 lipid uptake pathway.^170^ Further dietary studies in C57BL/6 mice and in vitro analysis of the murine 3T3‐L1 cell line revealed that FD promotes adipocyte differentiation and lipid accumulation through the C3a‐C3aR signaling pathway^171^ showing that, although FD may not be the predominant driver of obesity, it still has a clear role in metabolic activity.

The link between metabolic dysfunction, immunity, and non‐alcoholic fatty liver disease (NAFLD) is gaining greater interest, as NAFLD becomes the predominant global cause of cirrhosis and hepatocellular carcinoma.^172^ As explored above, FD deficiency is associated with altered adipocyte differentiation and increased hepatic lipid accumulation. In addition to this, other complement knockout mouse strains have been reassessed in the context of possible metabolic and liver dysfunction.^72^, ^95^, ^173^, ^174^ These recent data have implicated properdin in the control of obesity‐related liver and kidney disease, with *Cfp* ^−/−^ mice showing increased body weight, decreased expression of C5a like receptor 2 (C5L2 or C5aR2), decreased insulin sensitivity, obesity‐associated glomerulopathy, and increased hepatic triglyceride.^173^

### 1.9 Age‐related macular degeneration (AMD) and the AP/AL in mouse

Complement activation plays a role in AMD. Initial evidence of the involvement of complement activation in AMD came from the observation that drusen were found to contain various components of the complement system including C3, C5, and C5b‐9.^175^, ^176^, ^177^ The identification of a single nucleotide polymorphism (rs1061170—Y402H) in FH as a major susceptibility factor for AMD^178^, ^179^, ^180^, ^181^, ^182^, ^183^ and strong association of variants in complement genes, including *CFB*, *CFI*, and *C3*, with AMD has confirmed a role for the AP/AL. Although patients with AMD do exhibit higher serum levels of activation products C3d, Ba, C3a, C5a, SC5b‐9; C3, C4, FB, and FD, allowing for a systemic impact of genetic changes on AMD, local complement activation and the correct regulation of that activated complement are being described as critical to disease progression.^175^, ^184^, ^185^, ^186^ Whether the normal regulation of the AP/AL is required in the eye or systemically is still hotly debated.^187^

With these findings came an urgency to model complement dysfunction in the mouse. While mice do not possess a macula, it has been proposed that the retinal structures of the mouse eye are similar to those of the peripheral retinas in humans, and that they can exhibit the characteristics of dry AMD.^188^ Interestingly, analysis of gene expression profiles from young and old mice have shown that the RPE/choroid in the aged mouse becomes an immunologically active tissue, with complement being identified as the 2nd to top “pathway” using Ingenuity pathway analysis (behind leukocyte extravasation signaling which arguably could be a result of the C3a/C5a axis).^189^ C3 levels were reportedly ninefold higher in aged RPE/Choroid than in young mice, with all components of the AP/AL upregulated. These data suggest that this key component of the ageing eye in AMD is conserved across the species and validates use of mice for testing certain hypothesis around the pathogenesis of AMD.

### 1.10 The *Cfh* ^−/−^ mice and AMD

As noted above, the fact that gene polymorphisms in *CFH* associate so strongly with AMD in man^178^, ^179^, ^180^, ^181^, ^182^, ^183^ led to an investigation of the *Cfh* ^−/−^ mice for evidence of an AMD phenotype. Analysis of eyes from 2‐year‐old *Cfh* ^−/−^ mice indicated an accumulation of C3 in the neural retinal and a thinning of the Bruch's membrane, associated with disorganization of cellular organelles (mitochondria) in the RPE, providing additional data to support a role for the AP/AL in eye damage in aged mice.^190^ Subsequent functional and anatomical analysis of the eye in *Cfh* ^−/−^ mice revealed evidence of retinal damage,^190^, ^191^ a correlation with changes in oxidative stress,^192^ and mitochondrion dysfunction,^193^ all factors that are considered to lay the ground for AMD, as well as abnormalities in the retinal vasculature.^192^ Increased expression of C1q, C3, and FB but failed upregulation of CD59 were also noted in the eyes of *Cfh* ^−/−^ mice as they age.^194^ Finally, *Cfh* ^−/−^ mice held in a barrier (specific pathogen free) versus an open environment indicated that the additional stress, and presumably systemic complement activation associated with the open environment, was important for the development of retinal pathology noted in these mice.^195^ The eye‐disease in *Cfh* ^−/−^ mice is therefore considered to represent early AMD (particularly, as they do not show progression to geographic atrophy or choroid‐neovascularization).

The role of complement in eye disease was underlined in the human APOE4 knocked‐in (to the *APOE* gene) mouse. These animals, when aged and placed on a high‐fat, cholesterol‐enriched (HFC) diet, recapitulate many aspects of the human AMD phenotype, including RPE cell pathology, visual function deficits, and formation of sub‐RPE deposits, including significant deposition of C3.^196^ Backcross of hAPOE4 mice onto the *Cfh* ^−/−^ background (with HFC diet) was reported to generate an aggravated disease while backcross onto the sCrry expressing mice (with HFC diet) ameliorated disease, confirming that in this model of AMD the AP/AL played a distinct role in the eye damage.^197^

With the understanding that pathological outcomes similar to man were achievable in the eyes of mice that had altered AP/AL components, several studies were undertaken with the aim to tease apart the pathophysiological relevance of the FH Y402H variant. This SCR7 variant is in an area of FH important for “self” cell surface interactions, binding to C‐reactive protein and immune evasion by several pathogens.^198^ In one approach, transgenic mice were developed which expressed a chimeric FH protein (consisting of mouse (m)FH short consensus repeats (SCR) 1‐5, human FH SCR6‐8 and mFH SCR 19‐20; with the human SCR 6‐8 expressing Y at “402” or H at “402”) under control of an ApoE promoter.^199^ The selected mice, when bred onto the *Cfh* ^−/−^ background, were found to have normal C3 levels, suggesting these minimal FH constructs were functional, as might be expected from subsequent data on minimal FH constructs.^58^, ^65^, ^104^ The results from this model suggested a role for control of the AP/AL in macrophage activation with some additional evidence of the transgenic mice developing clinically visible drusen‐like sub‐retinal deposits by 12 months of age. However, this was irrespective of the amino acid at 402 and most marked in the absence of mFH potentially suggesting that function of the transgenes was not completely restoring AP/AL balance completely or appropriately. In a subsequent transgenic approach, using BAC clones to transfer full‐length human FH with either H402 or Y402 and backcross to the *Cfh* ^−/−^ stain, humanized CFH‐Y and CFH‐H mice (as they were denoted) were generated.^107^ Again, the H402 and the Y402 variants partially restored eye function; analysis of the eyes of mice expressing either human variant alone confirmed the function of hFH to regulate the AP/AL. However, no light was shed on the functional consequences in vivo of the human H402Y substitution, which is perhaps unsurprising given the aforementioned variability between orthologues that likely subsumes minor (potentially critical) differences between the two AMD‐linked human variants. In subsequent studies, *Cfh* ^*+/−*^ were favored, as with advanced age, and use of high‐fat diet, these mice developed sub‐RPE deposit formation which lead to complement activation and ultimately eye pathology which was considered more consistent with AMD.^200^

## 2 EYE DAMAGE/AMD STUDIES WITH *C3* ^*−/−*^ AND *C3aR* ^*−/*−^ MICE

From as early as 1985, we have been aware that components of cigarette smoke can trigger activation of AP/AL in normal human serum (NHS), possibly modifying C3 in such a way to prevent FH from binding;^201^ providing an environmentally modified C3 which is similar or more profound than that of the R102G, K155Q, or P314L genetic variants associated with AMD.^202^, ^203^, ^204^ In a mouse model designed to replicate this environmental cause of AMD,^148^ much of the phenotype was reversed by removal of FB and in later studies use of CR2‐FH or a C3a receptor antagonist were also able to prevent smoke exposure‐induced eye pathology.^205^ These data mirrored findings in a laser‐induced CNV model of eye pathology where CR2‐FH provided 60% of the level of protection noted in *Cfb* ^−/−^ mice in this model.^131^

Interestingly, studies with *C3* ^*−/−*^ animals suggest that systemic and local levels of C3 are critical for progression of inflammatory eye disease.^206^ Furthermore, in a light‐induced model of progressive retinal degeneration in rats, C3 was detected in retinal macrophages and the sub‐retinal space, particularly at the margins of the emerging lesion, suggesting a similar role in atrophic AMD.^207^

In other studies, the flip side of the AP/AL was noted, in that mice lacking C3aR were found to develop early onset and progressive retinal degeneration, linking a role in C3a generation to normal retinal function.^208^ The authors also note visual dysfunction in the *C3* ^−/−^ mice (B6;129S4‐C3^tm1Crr/J^) at 12 months old, findings that are largely supported above.^206^ The changes in the eye mapped to changes in NF‐kB activation and caspase‐3, and suggested C3aR signaling could be important in protecting retinal cells from light‐induced retinal degeneration. Further links to metabolic changes were noted by the fact that levels of lysophosphatidylcholine were significantly reduced in plasma from *C3aR* ^−/−^ (and *C3aR* ^−/−^ *C5aR* ^−/−^) mice.^208^ The authors noted that retinal cells likely require a certain level of complement activity for normal function,^208^ which given the data emerging from animal modeling of other neurological conditions^209^, ^210^ is probably correct. Whether this requirement for C3 and C3a (the AP/AL) in normal homeostatic functions has consequences for the long‐term use of complete complement inhibition in the treatment of human disease remains to be seen. However, it is clear that both too little and too much C3 activation is associated with retinal degeneration, suggesting that balanced C3 activation is required for normal eye health.

## 3 TESTING ADENO‐ASSOCIATED VIRUS (AAV) GENE THERAPY IN MOUSE EYES

One of Professor Peter Lachmann's legacies will of course be his drive to use FI as therapeutic, and with Prof's David Kavanagh and Andrew Lotery was a scientific founder of Gyroscope (now part of Novartis). They aimed to provide precision medicine treatment of patients with AMD via FI gene therapy. One of the supporting studies towards their current clinical trial (open‐label Phase I/II FOCUS clinical trial, NCT03846193, read out in 1st Quarter 2024) was the testing of AAV constructs carrying the human *CFI* sequence (not codon optimized) via a sub‐retinal injection of mouse eyes.^211^ Western blot and histological analysis clearly showed that FI was predominantly expressed in the RPE and photoreceptors. Furthermore, their analysis confirmed that the secreted FI in vitreous humor was able to cleave C3b in fluid phase assays confirming it was folded and processed normally, all of which gave the authors confidence that such an approach could be highly beneficial in man. Further, mouse modeling in the laboratories of Barbel Rohrer (using the mouse model of passive smoke inhalation) and AAV gene therapy with CR2‐FH, indicated the importance of control of the AP/AL basal to the RPE which the authors argued strongly for delivery of anti‐complement therapy to the RPE or outside the blood‐retina barrier.^155^

### 3.1 Closing statement

**FIGURE 2:**
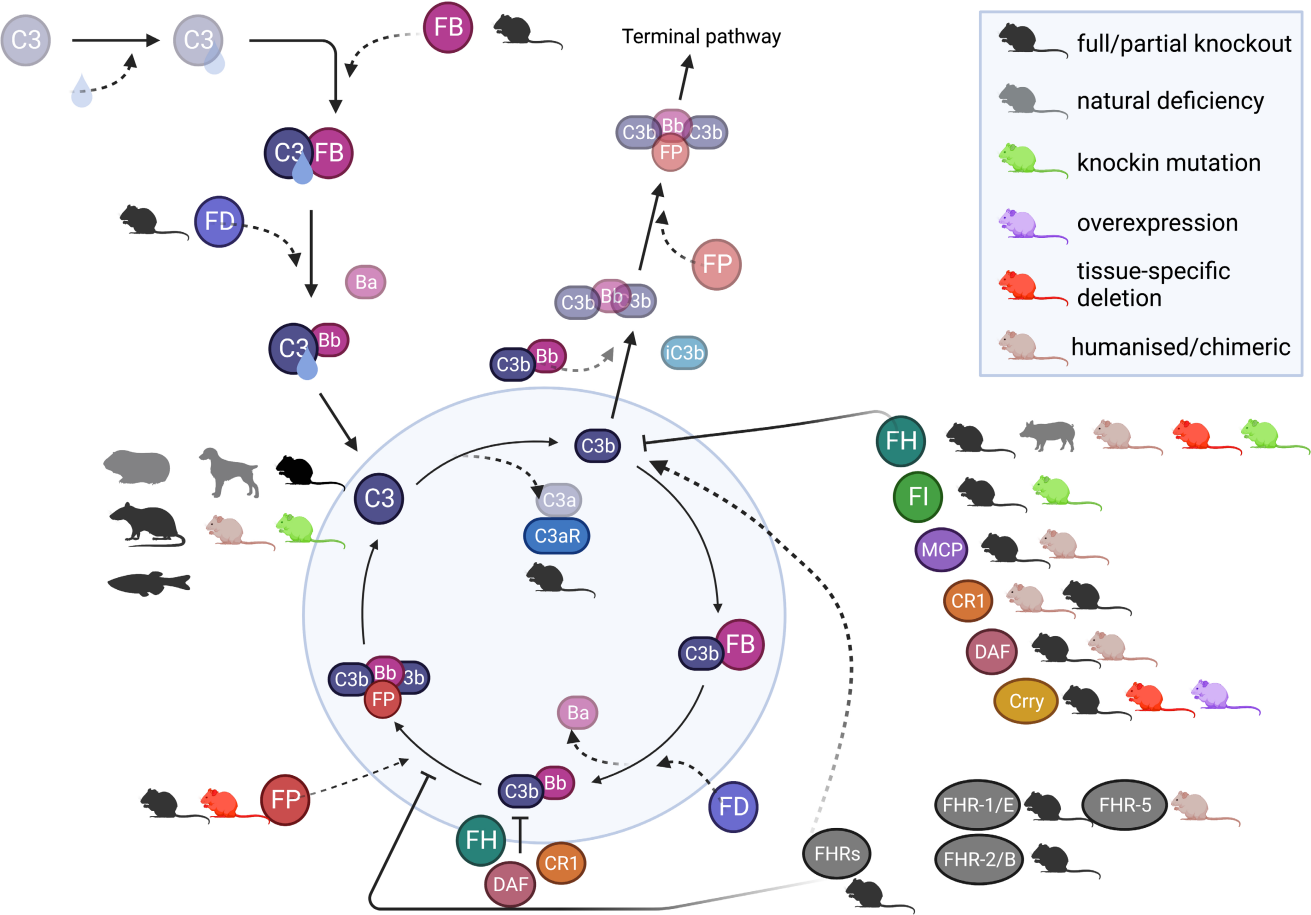
Naturally occurring complement deficient (gray) animal models have been available for over a hundred years, with C3‐deficient guinea pigs and dogs first described in 1919 and 1981, respectively. The predominant model species, however, is the laboratory mouse and knockout strains (black) have been created for almost every component in the AP/AL; over expression of Crry (Violet) allowed potential therapeutic approaches to be modelled. Advancing technology has allowed the more nuanced approach of tissue‐specific gene deletion (red). Knock‐in mutations (green) in FH, FI, and C3 have facilitated the study of human diseases and the development of humanized mice (brown) now allow in vivo drug testing against human targets.

Deletion of whole molecules/genes (i.e., *DAF* ^−/−^, *Cfh* ^−/−^, and *C3* ^−/−^ mice) as described herein, has been/is highly informative but it is rare in man. As noted, point mutations and polymorphisms are sufficient to increase human disease susceptibility; they may have only subtle effects at a molecular level but such variations can have profound and lasting effects on immunity. To us, this fits squarely with Chaos Theory and the Butterfly Effect or as Edward Lorenz put it “For those systems with bounded solutions, it is found that non‐periodic solutions are ordinarily unstable with respect to small modifications, so that slightly differing initial states can evolve into considerably different states”.^212^ How current complement‐modulating therapies, and the many proposed future drugs en‐route to the clinic act on the complement system across a lifetime in combination with these subtle genetic variations is still unknown. This is a matter of growing consequence and it is here that animal modeling provides potentially the best and only current option to try to evaluate these risks in a holistic manner. By exploiting the plethora of successful models of complement hypo and hyper‐activity (see Figure 2 and Table 3), we as researchers are empowered to dissect multiple facets of the role of complement in tissue/immune/organ homeostasis as well as the inevitable responses of the immune system to our concerted efforts to therapeutically correct unwanted complement activity. Our understanding of the alternative pathway and the integral amplification loop derived from many decades of animal modeling has driven rational design of complement therapeutics and other immune therapies. This is a legacy that was initiated by the studies of Prof Lachmann and others, who defined the ground from which we have built our models on but we would hope the “structures” we build have been far more enlightening than could have been predicted when some of us started down this path.

**TABLE 3:** Animal models used to illuminate the role of the AP/AL in disease

Modification to genome	Targeting approach	Target site	Comments	Reference
*CFD* gene disrupted	HR—KO	exon 3 replaced with Neo^R^ gene	No AP/AL function	47
*CVF* gene introduced	Tg	random insert	consumptive C3 depletion	49
*CFHR‐1* disrupted	C/C9	FHR‐E exons 1‐5 deleted by guide RNAs	reduced LPS mediated Complement activation	55
*CFH* disrupted	HR‐KO	exon 3 replaced with Neo^R^ gene	consumptive C3 depletion—Mouse model of C3G	57
Human C3 Knock‐in	VT	Exons 1 through 41 mC3 replaced with the human C3 gene and 5 prime regulatory elements	consumptive hC3 depletion—a model of C3G	59
*DAF* gene disrupted	HR‐KO	exon 1‐3 plus ~1kb 5 prime flanking region replaced with Neo^R^ gene	step towards a mouse PNH model	61
*CFB* gene disrupted	HR‐KO	exon 3‐7 replaced by Neo^R^ gene	No AP/AL function	74
*CFI* gene disrupted	HR‐KO	exon 4 was replaced by the Neo^R^ gene	Consumptive C3 depletion	77
*itgam* gene disrupted	HR‐KO	exon 1 replaced with Neo^R^ gene	essentially normal	80
*Cr1l* gene disrupted	HR‐KO	exon 5 replaced with Neo^R^ gene	embryonic lethal	88
*C3* gene disrupted v1	HR‐KO	exon 1, replaced by Neo^R^ in reverse orientation	intracellular C3 detected	89
*C3* gene disrupted v2	HR‐KO	RRR linker in Pro‐C3 replaced with Neo^R^ gene	most commonly used C3 KO	90
*CFP* gene disrupted v1	HR‐KO	exon 3‐7 replaced by Neo^R^	essentially normal	93
*CFH* gene altered	HR‐KI	Stop codons added to SCR19 of CFH	Termed FH^m/m^; essentially FH deficient—aged model of C3GN	95
*CFP* gene disrupted v2	HR‐cKO	LoxP sites flanking exon 5 and 6	unintentional properdin knockout line	96
*CFH* gene altered	HR‐cKO	LoxP sites flanking exon 2 and 5	hepatocyte FH deficiency on backcross with Alb‐Cre mouse	99
Humanized *CFH* locus	C/9	3 and 5 prime of entire mouse FH‐FHR locus	mice devoid of all FHRs and or FH and FHRs—AAV delivery of human proteins	104
human FH dimer construct introduction	AAV	Liver specific transduction	Gene therapy protective in mouse model of FHR5 nephropathy	104
Humanized *CFH*	Tg (BAC)	random insert	402H and 402Y variants generated	107
Truncated CFH	Tg	random insert	First mouse model of aHUS (no longer available)	113
Point mutant in *CFH* gene	HR‐ KI	FH 1206W changed to R	Mouse model of vascular thrombosis, TMA and aHUS	114
Point Mutant in *C3* gene	HR‐ KI	C3 1115D changed to N	Mouse model of TMA and aHUS (C3 GOF)	119
*CFD* gene disrupted	C/C9	exon 1 to exon 5 was deleted using two target sequences	protective against HFD‐induced liver damage	170
chimeric FH	Tg	Human SCR6‐8 introduced into mouse FH	402H and 402Y variants generated	
*C3aR* gene disruption	HR‐KO	part of exon 1 replaced with Neo^R^ gene	essentially normal; altered immune profiles	208
*C5aR* gene disruption	HR‐KO	entire coding region replaced with Neo^R^ gene	essentially normal; altered immune profiles on challenge	208
hFI introduction	AAV	RPE targeted	paved way to human clinical trials	211
hCR2‐FH construct introduction	AAV	RPE targeted	Importance of local RPE gene target for function in eye	155

*Notes*: Alb‐Cre, albumin promoter controlled Cre recombinase expression; AP/AL, alternative pathway of complement activation/amplification loop; AAV, adeno‐associated virus transduction; aHUS, atypical hemolytic uremic syndrome; BAC, bacterial artificial chromosome; C/C9—clustered regularly interspaced short palindromic repeats (CRISPR)/CRISPR‐associated protein 9(Cas9); *CFB*, complement factor B gene; *CFD*, complement factor D gene; *CFI*, complement factor I gene; *CFH*, complement factor H gene; CFHR‐1, complement factor H related ‐1 gene; FHR‐E Factor H related protein E; *CFP*, properdin gene; C3G, C3 glomerulopathy; C3GN, C3 glomerulonephritis; C3aR, C3a receptor gene; C5aR, C5a receptor gene; CR2‐FH, 4 N‐terminal SCRs of mouse complement receptor 2 (residues 1‐257 of mature protein; GenBank accession number M35684) linked (natural linker amino acids KIEL) to the five N‐terminal SCRs of mouse factor H (residues 1‐303 of mature protein; GenBank accession number NM009888); *CVF*, cobra venom factor; DAF, decay accelerating factor (CD55); *itgam*, Integrin alpha M gene; HFD, high‐fat diet; hFI, human Factor I; HR, Homologous recombination; GOF, gain‐of‐function; KO, gene knockout; KI gene knock‐in; LPS, lipopolysaccharide; LoxP, locus of x‐over‐P1; Neo^R^, neomycin resistance gene. PNH, paroxysmal nocturnal hemoglobinemia; RPE, retinal pigment epithelial cells; SCR, short consensus repeat; Tg, transgenic; TMA, thrombotic microangiopathy.

## CONFLICT OF INTEREST

KJM was a scientific consultant and provided research for Gemini Therapeutics. KJM has also received research funding from Qualasept, Catalyst Biosciences and Idorsia.

## Data Availability

Data sharing not applicable to this article as no datasets were generated or analysed during the current study
